# Endothelial Dysfunction and Cardiovascular Disease: Hyperbaric Oxygen Therapy as an Emerging Therapeutic Modality?

**DOI:** 10.3390/jcdd11120408

**Published:** 2024-12-19

**Authors:** Tanja Batinac, Lara Batičić, Antea Kršek, Danijel Knežević, Emanuela Marcucci, Vlatka Sotošek, Božena Ćurko-Cofek

**Affiliations:** 1 Department of Clinical Medical Sciences I, Faculty of Health Studies, University of Rijeka, Viktora Cara Emina 2, 51000 Rijeka, Croatia; tanjabatinac@net.hr (T.B.); emanuela.marcucci@uniri.hr (E.M.); 2 Department of Underwater and Hyperbaric Medicine, Clinical Hospital Center Rijeka, Tome Strižića 3, 51000 Rijeka, Croatia; 3 Department of Medical Chemistry, Biochemistry and Clinical Chemistry, Faculty of Medicine, University of Rijeka, Braće Branchetta 20, 51000 Rijeka, Croatia; lara.baticic@uniri.hr; 4 Faculty of Medicine, University of Rijeka, Braće Branchetta 20, 51000 Rijeka, Croatia; antea.krsek@student.uniri.hr; 5 Department of Anesthesiology, Reanimatology, Emergency and Intensive Care Medicine, University of Rijeka, Braće Branchetta 20, 51000 Rijeka, Croatia; danijel.knezevic2@uniri.hr; 6 Department of Physiology, Immunology and Pathophysiology, Faculty of Medicine, University of Rijeka, Braće Branchetta 20, 51000 Rijeka, Croatia; bozena.curko.cofek@medri.uniri.hr

**Keywords:** endothelium, endothelial dysfunction, endothelial glycocalyx, hyperbaric oxygen therapy, cardiovascular diseases

## Abstract

Maintaining the physiological function of the vascular endothelium and endothelial glycocalyx is crucial for the prevention of cardiovascular disease, which is one of the leading causes of morbidity and mortality worldwide. Damage to these structures can lead to atherosclerosis, hypertension, and other cardiovascular problems, especially in individuals with risk factors such as diabetes and obesity. Endothelial dysfunction is associated with ischemic disease and has a negative impact on overall cardiovascular health. The aim of this review was to comprehensively summarize the crucial role of the vascular endothelium and glycocalyx in cardiovascular health and associated thrombo-inflammatory conditions. It highlights how endothelial dysfunction, influenced by factors such as diabetes, chronic kidney disease, and obesity, leads to adverse cardiovascular outcomes, including heart failure. Recent evidence suggests that hyperbaric oxygen therapy (HBOT) may offer therapeutic benefits in the treatment of cardiovascular risk factors and disease. This review presents the current evidence on the mechanisms by which HBOT promotes angiogenesis, shows antimicrobial and immunomodulatory effects, enhances antioxidant defenses, and stimulates stem cell activity. The latest findings on important topics will be presented, including the effects of HBOT on endothelial dysfunction, cardiac function, atherosclerosis, plaque stability, and endothelial integrity. In addition, the role of HBOT in alleviating cardiovascular risk factors such as hypertension, aging, obesity, and glucose metabolism regulation is discussed, along with its impact on inflammation in cardiovascular disease and its potential benefit in ischemia–reperfusion injury. While HBOT demonstrates significant therapeutic potential, the review also addresses potential risks associated with excessive oxidative stress and oxygen toxicity. By combining information on the molecular mechanisms of HBOT and its effects on the maintenance of vascular homeostasis, this review provides valuable insights into the development of innovative therapeutic strategies aimed at protecting and restoring endothelial function to prevent and treat cardiovascular diseases.

## 1. Introduction

In recent decades, it has become evident that maintaining the physiological function of the vascular endothelium and endothelial glycocalyx is of great importance for the prevention of cardiovascular disease, which is a leading cause of mortality and morbidity worldwide. The protection and restoration of endothelial function and the integrity of the endothelial glycocalyx are of crucial importance in patients with cardiovascular risk factors to avoid the development of cardiovascular disease and possible thrombo-inflammatory conditions. Various factors, such as diabetes, chronic kidney disease, atherosclerosis, ischemia/reperfusion injury, dysmetabolic vascular disorders, and obesity, can contribute to damage and degradation of endothelial cells and the endothelial glycocalyx. Changes in the vascular wall subsequently lead to cardiovascular diseases such as coronary heart disease, cerebrovascular diseases, and peripheral artery diseases [1,2,3,4] and alter their outcomes [5]. Additionally, effects on cardiac metabolism and diastolic dysfunction lead to heart failure.

In recent years, there has been increasing evidence of a potentially beneficial effect of hyperbaric oxygen therapy (HBOT) on various cardiovascular risk factors as well as on cardiovascular disease. HBOT is a non-invasive treatment based on exposure to 100% oxygen at elevated atmospheric pressure. According to the Undersea and Hyperbaric Medical Society (UHMS), this pressure should be 1.4 atmospheres or more, although all current UHMS-approved indications for HBOT require patients to breathe 100% oxygen at a pressure of at least 2 atmospheres [6,7]. The therapeutic basis of HBOT relies on three factors: breathing 100% oxygen favors diffusion into hypoxic tissue; the concentration of oxygen in the blood increases as the pressure increases according to Henry’s law, which states that the amount of dissolved gas in a liquid is directly proportional to its partial pressure; and the size of gas bubbles in the blood decreases according to Boyle–Mariotte’s law and Henry’s law [6,8]. HBOT increases the percentage of oxygen dissolved in plasma [9] leading to hyperoxemia and hyperoxia that do not affect hemoglobin levels and is used in the treatment of various pathological conditions [10,11,12,13]. HBOT exerts its beneficial effects by promoting angiogenesis, antimicrobial properties, and immunomodulatory effects, improving antioxidant defenses, and stimulating stem cells [13,14]. For clinical purposes, HBOT is usually administered at a pressure between 2 and 3 atmospheres absolute (ATA), most commonly at 2.4 ATA. Above this pressure, oxidation products accumulate, and antioxidants become saturated, leading to oxidative damage [9]. Prolonged exposure to high oxygen concentrations and failure to counteract oxidative stress can lead to oxygen toxicity and systemic changes resulting in seizures, pulmonary insufficiency, and retinopathy of prematurity. Furthermore, according to Boyle’s law, increased ambient pressure in the HBOT chamber compresses air-filled spaces in the body. Rapid pressure changes can lead to tissue damage like ear, sinus, and pulmonary barotrauma, if equalization of pressure does not occur properly [6]. In addition, HBOT can enhance insulin sensitivity and increase glucose uptake by cells. This may lead to a rapid drop in blood sugar levels, especially in patients on insulin therapy [8]. Moreover, hyperoxia can transiently affect the lens and cornea. Over time, repeated HBOT sessions can lead to changes in the refractive index due to oxygen-induced modifications in the lens [9,13].

The aim of this review was to comprehensively summarize the current understanding of the molecular mechanisms of HBOT and its impact on the maintenance of vascular homeostasis, with particular emphasis on the vascular endothelium and the endothelial glycocalyx. While highlighting the positive therapeutic effects of HBOT, this review also underscores the importance of considering its potential side effects, which can arise from hyperoxia and pressure-related changes. By providing a balanced and detailed examination of both the beneficial and adverse outcomes associated with HBOT, this review aimed to offer a holistic perspective of HBOT application from different points of view.

## 2. The Vascular Endothelium and the Endothelial Glycocalyx Structure and Function

The endothelial cells of the vascular endothelium, which is lined by endothelial glycocalyx, play an important role in the maintenance of vascular homeostasis. They are involved in the maintenance of vascular integrity and vascular tone [15,16], nitric oxide (NO) production [16,17], mechanotransduction of extracellular signaling [16,18], regulation of the coagulation process and fibrinolysis, support of anti-inflammatory properties, and mediation of leukodiapedesis [15,19]. In addition, endothelial cells secrete cytokines, chemokines, and various growth factors in response to noxious substances, regulate their activity and the inflammatory response, inhibit the binding of leukocytes to the endothelial surface, and impede antigen presentation and T cell activation [15,20].

The endothelial glycocalyx is a carbohydrate-rich layer of proteoglycans and glycosaminoglycans (GAGs) that lines the luminal surfaces of endothelial cells of all blood vessels, ranging from capillaries to large arteries and veins [5,15,21].

Proteoglycan’s core proteins are mainly syndecans and glypicans which are bound to the cell membrane. In contrast, perlecan, versican, decorin, biglycan, and mimecan are secreted as soluble proteoglycans [22]. Syndecans are present on the surface of almost all cells in the body. They are transmembrane proteins and therefore consist of three parts: the cytosolic C-terminal domain, the transmembrane domain, and the N-terminal extracellular domain. The extracellular part varies between the different members of the syndecan family, while the other two domains are highly conserved [23].

Four members of the syndecan family are known in mammals. Syndecan-1 is mainly found in epithelial and plasma cells, syndecan-2 in mesenchymal cells, and syndecan-3 in neural tissues, while syndecan-4 is found in numerous cell types [24]. The extracellular domain of the protein core of syndecan binds various GAGs, which consist of negatively charged, repeating disaccharide chains. To date, there are five types of GAGs: hyaluronic acid, heparan, chondroitin, keratin, and dermatan sulfates. Among them, heparan sulfate (HS) is the most dominant [5]. Thus, syndecan-1 and -3 can have HS and chondroitin sulfate chains, while syndecan-2 and -4 only have HS in the extracellular domain [23].

**Figure 1:**
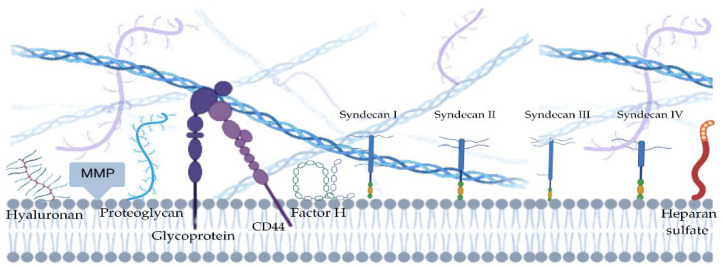
Schematic representation of endothelial glycocalyx structure. MMP–matrix metalloproteinases.

Since the cytoplasmic domain of syndecan is in contact with protein kinase C, it can initiate the activation of intracellular signaling pathways [25]. It also interacts with the cytoskeleton and is involved in endocytosis and exosome biogenesis [26]. Six members of the highly conserved glypicans (glypican-1 to glypican-6) form another group of the proteoglycan family. They are bound to the cell surface and each glypican has two to four HS chains inserted at the C-terminal domain, close to the cell membrane (Figure 1).

Together with the core proteins, these chains mediate the function of the glypicans. Studies have shown that glypicans modulate cell signaling pathways by regulating the binding of growth factors to signaling receptors [27]. Glypicans are also important for the function of the nervous system. They are part of the synapse-forming protein complex and are involved in neuron guidance and migration [28]. Proteoglycans and glycoproteins form a network into which soluble molecules from plasma (such as albumins and orosomucoid) or the endothelium are incorporated, contributing to the thickness and permeability of the endothelial glycocalyx [29]. The thickness and structure of the endothelial glycocalyx differ significantly between different vascular beds [30,31] and correlate with its function and integrity maintained by physiological blood flow, depending on the exposure of endothelial cells to shear stress [32]. For example, the endothelial glycocalyx in pulmonary capillaries is very thin to facilitate gas exchange between the alveoli and the pulmonary circulation [22]. Reduced thickness and altered structure of the endothelial glycocalyx have been associated with vascular dysfunction and disease [15]. Alteration of the integrity of the endothelial glycocalyx significantly disrupts vascular homeostasis, as the specific interaction of various plasma proteins involved in this process, such as complement-regulating proteins like factor H (fH) and C1 inhibitor and various regulatory proteins of the coagulation system like antithrombin III (ATIII), depend on binding to HS-binding domains [15,33]. Significant glycocalyx damage and the release of syndecans or HS have been demonstrated in post-cardiac arrest syndrome, myocardial infarction, atherosclerosis, stroke, sepsis, and ischemia–reperfusion injury [5,15,34]. Damage to the glycocalyx has been shown to lead to increased leukocyte rolling and adhesion [15,35], increased vascular permeability [36], coagulation [15,19], and altered vascular tone [17]. In addition, soluble components of the glycocalyx may trigger the release of pro-inflammatory cytokines [37].

Vascular shear stress is an important regulator of NO production, upregulating the expression of endothelial nitric oxide synthase (eNOS) when HS structures are preserved [15,16,18]. It has been suggested that the shear-induced force acting on membrane-anchored proteoglycans such as syndecan and glypican-1 leads to phosphorylation of eNOS, which activates and increases NO production [38]. Glypican-1 inactivation has been shown to block eNOS activation [38,39]. In areas of oscillatory shear stress, turbulent blood flow damages the endothelial surface and removal of glycocalyx components. Enzymatic shedding of HS and sialic acids from cultured aortic endothelial cells has been shown to block shear-induced NO expression and impair flow-mediated vasodilation [15,39,40].

In addition, mechanotransduction of shear stress has been shown to regulate various intracellular signaling pathways involved in the control of thrombosis and fibrinolysis, as well as inflammation, and activate various transcription factors involved in cell survival and in the regulation of monocyte migration and infiltration [15]. Depending on the level of shear stress, it has been shown to increase the expression of syndecan-1 and syndecan-4 [15,41].

Endothelial cells physically control the migration of cells and molecules through the vessel wall and prevent vascular leakage. Endothelial junctions are considered the key players in maintaining vascular integrity, while the glycocalyx layer acts as a charge-selective barrier for the transport of molecules into the intra- and subcellular space [15]. Tight junctions regulate cellular permeability and control exchanges between the luminal and basolateral cell membranes [42], while adherent junctions maintain tissue structure and modulate cell specification and growth by transmitting various signals [43]. Studies have shown that vascular permeability in vivo is dependent on glycocalyx composition and endothelial cell and glycocalyx structure, as removal of HS from the endothelial glycocalyx layer has been shown to reduce the expression of the gap junction protein connexin [43], leading to increased vascular permeability [44].

The glycocalyx can regulate inflammation and blood coagulation through direct interaction with plasma proteins and circulating cells. This is mediated by the interaction of HS, which has been shown to have binding sites for various growth factors, cytokines, and chemokines as well as various plasma proteins with HS-binding domains [45]. The binding of HS to various growth hormones, such as fibroblast growth factor (FGF), vascular endothelial growth factor (VEGF) [46], and granulocyte–macrophage colony-stimulating factor (GM-CSF) [47], influences their stability, protects them from degradation, and facilitates intracellular signaling [15]. The glycocalyx performs various functions in maintaining vascular stability and enhances its function following the interaction of HS and plasma proteins that have binding sites that increase their function, such as ATIII, superoxide dismutase (SOD), and xanthine oxidase (XOD), which are involved in protection against oxidative stress [15].

## 3. The Vascular Endothelium and the Endothelial Glycocalyx in Atherosclerosis and Cardiovascular Disorders

Endothelial glycocalyx damage and degradation have been associated with the onset and worsening of various conditions and diseases, particularly cardiovascular disorders or associated risk factors, such as acute coronary syndrome [48,49], chronic heart failure [50], ischemia–reperfusion injury [51], atherosclerosis [52], stroke [53], diabetes [54], obesity [55], hypertension [52,56], dyslipidemia [56], coronary artery disease [52,57], and cardiogenic shock [58]. Cardiovascular risk factors for the development of atherosclerosis such as vascular aging, hypertension, obesity, and diabetes lead to damage and thinning of the endothelial glycocalyx and are associated with the progression of atherosclerotic diseases and the pathogenesis of cardiovascular events [4,5,15]. The initial stage of cardiovascular diseases is characterized by endothelial dysfunction and a reduced bioavailability of NO [59]. In addition, impaired endothelial glycocalyx has been associated with arterial stiffness, coronary microcirculatory dysfunction, and abnormal myocardial function in untreated hypertensive patients [60].

Endothelial dysfunction as a trigger for atherosclerosis shows HS degradation which leads to the significant release of pro-inflammatory cytokines and chemokines, and the production of reactive oxygen species (ROS), that, in conjunction with a decreased production of NO, promotes the progression of atherosclerosis [4,15,52,61]. Altered NO production highlights the complex relationship between impaired endothelial function, inflammation, and atherosclerosis in the pathogenesis of cardiovascular events [62,63]. Degradation of the endothelial glycocalyx, and consequently increased vascular permeability, can cause LDL cholesterol accumulation in the subendothelial space and trigger an inflammatory reaction that leads to the formation of atherosclerotic plaques [64].

Further promotion of endothelial dysfunction by inflammation is facilitated by the increased expression of adhesion molecules that recruit immune cells to the site of injury, further contributing to plaque development and progression [65]. In atherosclerosis, activated endothelial cells promote inflammation and migration of macrophages into the intima of the vessel and differentiation into macrophages that take up oxidized lipoproteins, leading to foam cell formation and thus plaque formation [64,65,66]. Over time, plaques can become unstable and lead to acute cardiovascular events such as myocardial infarction or stroke [67].

Furthermore, macrophage activation leads to increased expression of matrix metalloproteinases (MMPs), which additionally damage the endothelial glycocalyx and promote endothelial dysfunction [61,68]. A recent study in Sprague Dawley rats with endothelial dysfunction has shown that HBOT effectively reduces the expression of MMP-12, which plays a significant role in atherosclerosis and affects vascular tissue and thus could contribute to the prevention and treatment of atherosclerotic heart disease [61].

Intact HS proteoglycan structures have been shown to protect against atherosclerotic plaque development by binding to a proliferation-induced ligand (APRIL), thereby reducing lipoprotein retention, macrophage migration into the vascular intima, and plaque formation [69]. It has been suggested that partial carotid artery ligation in apolipoprotein E-deficient (ApoE-/-) mice prone to atherosclerosis increases oscillatory shear and the production of microRNA-712, which inhibits the tissue inhibitor of MMP-3, leading to the development of atherosclerotic plaques [70,71], which has been associated with a complete deficiency of hyaluronan [72]. Degradation of hyaluronan and HS from the endothelial glycocalyx has been shown to inhibit shear-induced NO production [73].

In addition, vascular endothelial injury results in a pro-thrombotic and antifibrinolytic endothelial cell phenotype, predisposing the damaged site to thrombosis, and decreased NO production and release [74], which impairs endothelium-dependent vasodilation and leads to additional accumulation of lipoproteins.

Atherosclerotic plaque formation occurs most frequently at sites of impaired or reduced blood flow such as bifurcations and branch points [67] and is exacerbated by hypercholesterolemia leading to endothelial dysfunction mainly due to reduced bioavailability of NO [67]. It has been shown that impaired or reduced blood flow leads to increased damage to the endothelial glycocalyx, mainly through increased activity of sheddases, which are proteolytic enzymes that cause the detachment of glycocalyx components [75]. This is related to in vitro studies showing thinning of the endothelial glycocalyx in areas of impaired blood flow associated with atherosclerotic plaque formation [45] and increased levels of hyaluronic acid and syndecan-1 in the plasma of patients with coronary artery disease [48]. Treatment of apolipoprotein E-deficient mice with an inhibitor of hyaluronan synthesis and hyaluronan degradation via the CD44 molecule damaged endothelial function and promoted atherosclerosis [72]. Atherosclerosis-prone ApoE-/- mice fed with a high-cholesterol diet showed increased LDL deposition and greater apoptosis in the common carotid artery region associated with thinner endothelial glycocalyx [45]. In addition, a high salt diet has been shown to cause a detachment of syndecan-1 [76] and a reduction in the HS content of the endothelial glycocalyx [77]. Furthermore, in hypertensive patients, reduced glycocalyx thickness has been associated with vascular stiffness, an independent predictor of cardiovascular risk [60].

## 4. The Mechanism of HBOT

HBOT offers a multifaceted therapeutic approach by harnessing the combined effects of increased ambient pressure and elevated oxygen levels [6]. By significantly enhancing the amount of oxygen dissolved in plasma, HBOT ensures that oxygen can reach even hypoxic or ischemic tissues, where traditional hemoglobin-based oxygen delivery is impaired. This oxygen-rich environment facilitates cellular repair and supports energy production through restored mitochondrial oxidative phosphorylation [6,7,8,13,78]. A key benefit of HBOT is its ability to stimulate angiogenesis and neovascularization. The therapy promotes VEGF expression, aiding in the proliferation and migration of endothelial cells to form new blood vessels. This effect is partly mediated through the transient stabilization of hypoxia-inducible factor-1α (HIF-1α), which paradoxically persists under hyperoxic conditions. Additionally, NO production is enhanced through increased activity of eNOS, further supporting vascular growth and homeostasis [7,8,9,13,79].

HBOT also exerts profound anti-inflammatory effects by modulating immune responses. It suppresses the pro-inflammatory nuclear factor kappa B (NF-κB) pathway, reducing cytokine production, and shifts macrophage polarization from the inflammatory M1 phenotype to the reparative M2 phenotype, thereby aiding tissue recovery. These anti-inflammatory effects are complemented by HBOT’s ability to stimulate the mobilization of stem and progenitor cells, particularly through the activation of stromal-derived factor-1 (SDF-1) and CXCR4 signaling. This mobilization enhances tissue regeneration and repair [13,79].

The therapy also strengthens antimicrobial defenses by boosting the production of ROS and reactive nitrogen species (RNS), which enhance microbial killing by immune cells. Additionally, it creates an oxygen-rich environment unfavorable for anaerobic pathogens, contributing to its effectiveness against infections such as gas gangrene [10,78,79]. In wound healing, HBOT accelerates recovery by stimulating fibroblast proliferation, promoting collagen synthesis, and regulating MMPs to balance tissue remodeling. Similarly, in neurological recovery, it supports neuroprotection by improving oxygenation in ischemic brain tissues, reducing neuroinflammation, and enhancing neurogenesis through brain-derived neurotrophic factor (BDNF) signaling [10,13,78,79]. However, its intricate interplay of mechanisms underscores the importance of individualized therapeutic protocols to maximize benefits while minimizing risks [6,7,8,9,10,11,12,13].

A key to the effect of HBOT is the hyperoxic–hypoxic paradox [59,78]. It has been shown that exposure to intermittent hyperoxia can lead to beneficial effects in the cell. This process involves many cellular mechanisms induced by hypoxia [78,79]. Changes in oxygen levels due to intermittent 5 min air brakes every 20 min in the HBOT protocol increase the production, stability, and activity of HIF-1 [79,80,81]. Repeated intermittent hyperoxia due to air brakes or intermittent changes in oxygen levels that occur between daily sessions results in intermittent normoxia, perceived by tissues as hypoxia, that stimulates cellular protective mechanisms activating HIF-1 [14,78,79]. In addition, HBOT upregulates HIF-1 through ROS/RNS and the pathway of extracellular regulated kinases (ERK1/ERK2) [13,78].

HBOT-induced mitohormesis plays a key role in modulating monocyte and macrophage responses through controlled oxidative stress. The transient increase in ROS activates adaptive pathways, including the upregulation of Nrf2, which enhances antioxidant defenses, and the stabilization of HIF-1α, promoting cellular adaptation [13,78,79]. These effects drive macrophage polarization from the pro-inflammatory M1 phenotype to the reparative M2 phenotype, accompanied by increased IL-10 and reduced pro-inflammatory cytokines like TNF-α and IL-1β. Additionally, HBOT suppresses NF-κB activation, mitigating excessive inflammation. Through these mechanisms, HBOT regulates inflammatory responses, fostering tissue repair and regeneration [10,13,78,79].

During HBOT, the increased oxygen solubility in plasma under hyperbaric conditions combats hypoxia, maintains tissue viability reversibly damaged by hypoxia and ischemia, and restores microcirculation [13,79]. Hyperoxia increases the production of ROS and RNS [9,13,79]. ROS have multiple effects and play an important role in the organism by influencing apoptosis, cell signaling, synthesis of various growth factors, neovascularization, or immunomodulation and are involved in processes such as hyperglycemia, diabetes mellitus, protein aggregation, neurodegeneration, and cancer [9,13,79,82].

In addition to the beneficial effects, the accumulation of ROS and RNS can lead to metabolic disorders, endothelial dysfunction, acute pulmonary injury, and neurotoxicity due to uncontrolled production and/or reduced degradation, as well as cumulative oxidative damage such as lipid peroxidation, protein dysfunction, and DNA damage [9,13,83,84].

When ROS production exceeds antioxidant capacity, oxidative stress occurs. Mitochondria are a primary site for ROS production [85]. Studies have shown inconsistent results regarding the effects of HBOT on oxidative stress balance and mitochondrial function, mainly due to the different number of sessions, protocols applied, pressure, and duration of the session [9]. HBOT has been shown to increase ROS production and induce oxidative stress, suggesting negative effects in some cases and emphasizing its therapeutic properties in others. Breathing high concentrations of oxygen increases ROS and oxidative stress. ROS can damage cellular components such as lipids, proteins, and DNA which can lead to central nervous system (CNS) toxicity with symptoms like nausea, dizziness, vision changes, twitching, and in severe cases, seizures. Hyperoxic stimulation of neurons, particularly through calcium influx and excitatory neurotransmitter release, can contribute to neurotoxicity. [9,86,87]. Moreover, HBOT has been found to reduce mitochondrial function [88] or, on the contrary, to provide effective antioxidant protection by improving mitochondrial activity and increasing free radical scavenging [9,89,90]. The accumulation of ROS and RNS can lead to metabolic disorders, endothelial dysfunction, acute pulmonary injury, and neurotoxicity due to uncontrolled production and/or reduced degradation, as well as cumulative oxidative damage such as lipid peroxidation, protein dysfunction, and DNA damage [9,79,83]. Different forms of ROS can be inactivated by the action of various antioxidants [9]. Enzymatic antioxidants such as SOD, catalase (CAT), heme oxygenase 1 (HO-1), and thioredoxin- and glutathione-dependent peroxidase (GPx) and reductase(s) convert ROS into water or oxygen [91]. In addition, non-enzymatic antioxidants, and endogenous free radical scavengers, such as vitamin C, vitamin E, glutathione, melatonin, and β-carotene [92], reduce ROS levels by donating an electron to stabilize unstable reactive species [9]. Li et al. reported that HBOT demonstrates an antioxidant effect enhancing the activity of CAT and SOD, thereby reducing the production of free radicals generated during ischemia–reperfusion injury [93]. In addition, HBOT has been shown to increase antioxidant SOD and HO-1 activity, reversing the increased oxidative stress caused by radiation therapy [94].

**Figure 2:**
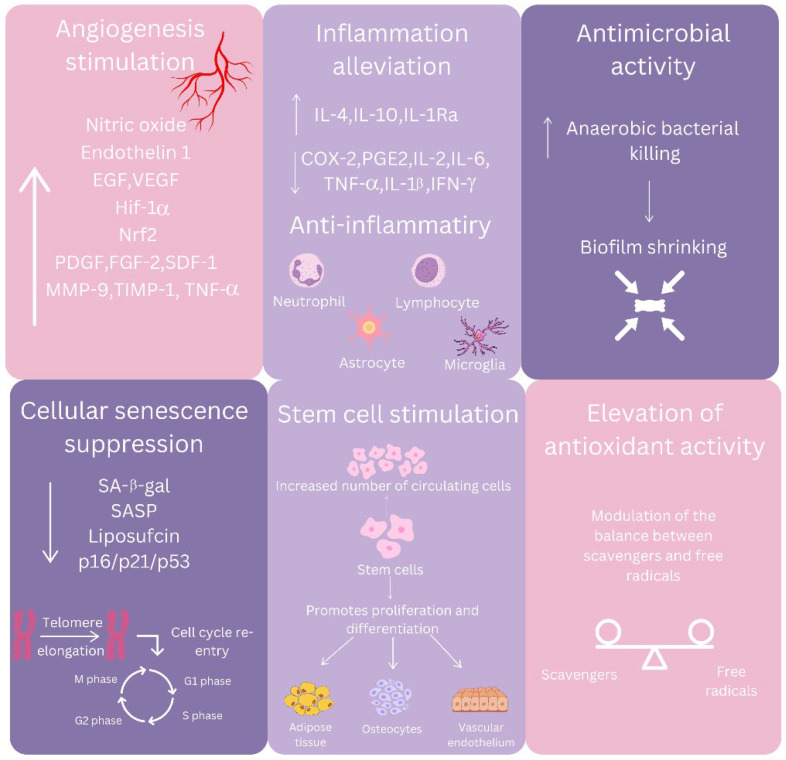
Key effects of a therapeutic intervention across six biological pathways: Angiogenesis stimulation encourages new blood vessel growth through molecules like nitric oxide (NO), vascular endothelial growth factor (VEGF), and growth factors (GFs) such as platelet-derived growth factor (PDGF-2) and fibroblast growth factor (FGF-2), which help repair and regenerate tissues. Inflammation alleviation reduces inflammation by increasing anti-inflammatory cytokines [e.g., interleukins (IL-4, IL-10)] and lowering pro-inflammatory molecules [e.g., cyclooxygenase-2 (COX-2) and tumor necrosis factor alpha (TNF-α)], aiding immune regulation and reducing tissue damage. Antimicrobial activity boosts the body’s ability to kill anaerobic bacteria and reduces biofilm formation, enhancing resistance to infections. Cellular senescence suppression slows down the aging process by downregulating markers of cellular senescence (e.g., senescence-associated β-galactosidase (SA-β-gal) and cellular senescence markers (p16/p21/p53)) and promoting telomere elongation, which helps cells avoid age-related dysfunction and re-enter the cell cycle. Stem cell stimulation increases the number of circulating stem cells, promoting their differentiation into various tissue types like adipose cells and osteocytes, thus supporting tissue regeneration and healing. Elevation of antioxidant activity enhances antioxidant defenses by modulating the balance between free radicals and scavengers, protecting cells from oxidative stress and damage, which is crucial for maintaining cellular health.

Hyperbaric oxygen has also been shown to have both positive and negative effects on the body acting through direct and indirect mechanisms [9,13,79,82] as summarized in Figure 2. Primary effects include correction of hypoxia, antimicrobial activity, and reduction in HIF-mediated activity, while secondary effects include reduction in inflammation, reduction in ROS, enhancement of healing, angiogenesis, and vasoconstriction, and attenuation of reperfusion injury [13,79,82].

Short-term (1 to 5 consecutive treatments) or single exposure to HBOT, generally, leads to a decrease in mitochondrial function, while long-term treatments (20–60 consecutive treatments) lead to a significant increase in mitochondrial function [9,13,95]. Various animal studies have shown that repeated HBOT treatments for more than 20 days have a positive effect on mitochondrial activity and metabolism [88,96,97]. An HBOT protocol involving repeated daily 90 min exposures (60 sessions, 5 days per week) with a 5 min air brake every 20 min has been shown to induce regenerative processes in non-healing wounds and certain brain injuries [98,99,100].

The difference in the effects and outcomes obtained after HBOT is explained by the concept of hormesis and is dose-dependent [101], suggesting that treatment with subtoxic doses of a toxicant could lead to adaptations to prevent damage [14,101,102]. Multiple sessions of HBOT have been shown to result in unchanged systemic levels of oxidative stress and signs of decreased ROS production in healthy young subjects [103], or even, as recently reported, oxidative stress levels might decrease after HBOT in middle-aged men [90]. HBOT leads to elevated ROS production as well as an adaptive response to ROS accumulation in the form of increased scavenger production, which is insufficient and gradual after limited exposure [78]. Mitochondrial respiration is reduced to mitigate oxidative stress due to increased ROS production [88], which is consistent with studies showing a reduction in mitochondrial activity after short-term HBOT and an initial increase in ROS [95].

**Figure 3:**
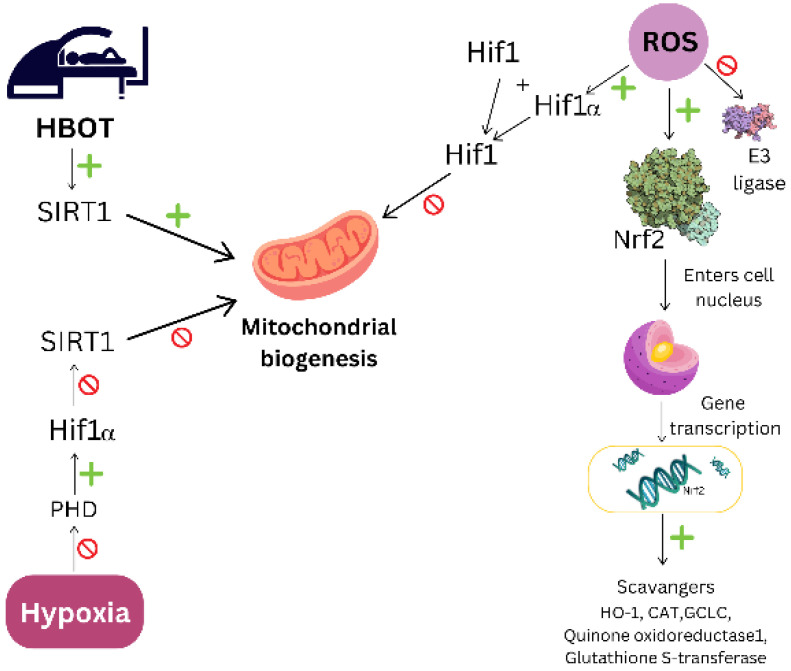
The tissue response to hypoxia and repeated intermittent hyperoxia during hyperbaric oxygen therapy (HBOT) results in a biphasic response and involves an accumulation of reactive oxygen species (ROS) alongside an enhanced cytoprotective antioxidant response. Hypoxia and intermittent HBOT promote the activation of hypoxia-inducible factor-1 (HIF-1), either by directly inhibiting prolyl hydroxylase domains (PHDs) or by increasing antioxidants that suppress PHD activity. During hyperoxia, ROS production increases, leading to the activation of HIF-1α, which conjugates with HIF-1β to stabilize HIF-1 in its active form. HIF-1, in turn, inhibits mitochondrial biogenesis. Increased mitochondrial consumption of NADH raises NAD+ levels, which activates SIRT1, improving mitochondrial biogenesis and inducing antioxidant defenses. As part of an adaptive mechanism, elevated ROS levels stimulate the production of endogenous scavengers, whose elimination half-life is significantly longer than that of ROS. Additionally, HBOT enhances antioxidant enzyme levels by activating transcription factors and gene expression via the nuclear factor erythroid 2-related factor 2 (Nrf2) pathway and its downstream targets, including heme oxygenase-1 (HO-1), NAD(P)H quinone dehydrogenase 1 (NQO-1), catalase (CAT), glutathione peroxidase (GPx), superoxide dismutase (SOD), and glutamate–cysteine ligase catalytic subunit (GCLC), while reducing pro-oxidant enzymes such as inducible nitric oxide synthase (iNOS) and gp91-phox.

As mentioned above, HBOT induces a biphasic response as presented in Figure 3: an accumulation of ROS is accompanied by an enhanced cytoprotective antioxidant response, which tends to be more pronounced after repeated exposures [9,14]. ROS and RNS also serve as signaling molecules in transduction cascades responsible for regulating cell signaling and cell survival, apoptosis, and proliferation through the upregulation of various factors such as nuclear factor erythroid 2-related factor 2 (Nrf2) and HIF-1, Sirtuin1 (SIRT1), VEGF, growth factors, and hormones [104,105]. A mechanism involved in tissue response to hypoxia and repeated intermittent hyperoxia during HBOT is summarized in Figure 3.

Accumulating data suggest that HBOT enhances antioxidant responses by upregulating Nrf2, a redox sensor and master regulator of cellular defenses against oxidative stress, by regulating downstream target genes such as HO-1, glutathione-S-transferase and quinone oxidoreductase, NAD(P)H quinone dehydrogenase 1 (NQO-1), CAT, GPx, SOD, glutamate–cysteine ligase catalytic subunit (GCLC), and others [89,106], which reduce ROS levels and their activation in response to oxygen partial pressure [9]. Respiration of 100% oxygen increases Nrf2 and upregulates Nrf2-regulated genes [9,107]. Normally, Nrf2 is degraded by the proteasome, but upon oxidative stress, it is stabilized and transported to the nucleus where it activates its known antioxidant response and activates target genes [104,105]. Tissue Nrf2 levels in diabetic ulcers have been shown to increase significantly after 25 sessions of HBOT compared to untreated patients [89]. In addition, HBOT enhances glycolysis by upregulating key enzymes such as hexokinase, phosphofructokinase, and pyruvate kinase. This metabolic shift supports energy production and reduces the oxidative burden by minimizing mitochondrial ROS production. Consequently, HBOT strengthens the cellular ability to regulate hypoxia-induced oxidative stress, ensuring improved adaptation, repair, and survival following ischemic or hypoxic injury [13,89].

Repeated intermittent hyperoxia exposures or long-term HBOT activates SIRT1, a key mitochondrial stimulator and important part of a cellular defense mechanism against oxidative stress [108], by increasing NAD+ levels from the hyperoxic state during HBOT [109]. This enhances mitochondrial biogenesis via acetylation of PGC-1α and induces antioxidant responses via deacetylation of forkhead box O3a (FOXO3a) [105]. On the other hand, hypoxia and apparent hypoxia during intermittent HBOT reduce NAD^+^ levels, which inhibits SIRT1 and decreases mitochondrial biogenesis and the effect of hyperoxia [9].

HIF-1 plays an important role in several processes that counteract hypoxia, such as red blood cell formation through the transcription of erythropoietin and blood vessel formation through the activation of VEGF [9,110]. HBOT-activated HIF-1 counteracts the promoting effect of SIRT1 on mitochondrial function [9,78,111]. Normally, prolyl hydroxylase domain (PHD) proteins recognize oxygen and destabilize HIF1, leading to its degradation, while hypoxia and intermittent HBOT increase activation of HIF-1, either by directly inhibiting PHDs or by increasing antioxidants that inhibit PHDs [9,59], inducing the transcription of genes involved in the adaptation of cells to low oxygen levels or responsible for promoting erythropoiesis, angiogenesis, and glycolysis that counteract hypoxia [59,86].

**Figure 4:**
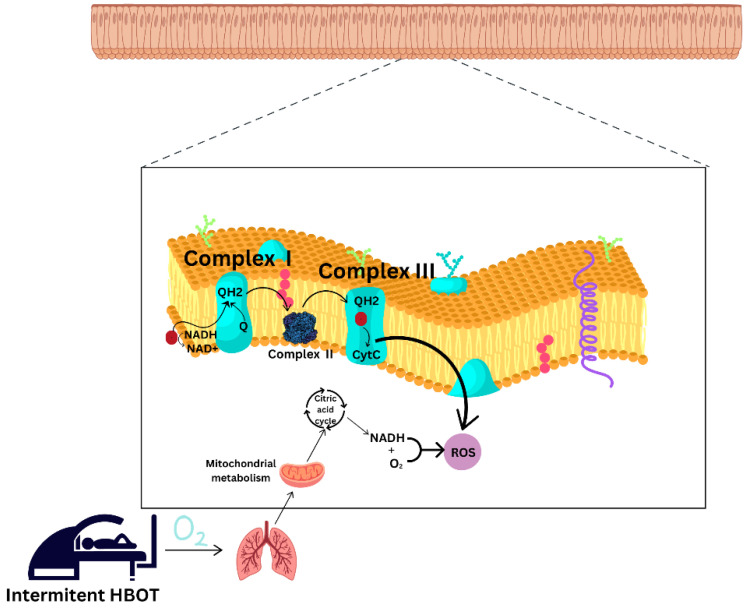
The effect of hyperbaric oxygen therapy (HBOT) on oxidative stress balance at the level of the mitochondrial membrane. In HBOT, oxygen from the lungs increases the content of oxygen dissolved in the plasma, resulting in tissue hyperoxia that boosts the citric acid cycle in mitochondria, increasing nicotinamide adenine dinucleotide (NADH) production, which can react directly with oxygen to produce reactive oxygen species (ROS). Increased ROS levels can produce more endogenous scavengers, with the elimination half-life being much longer than that of ROS. HBOT stimulates antioxidant defenses via activation of nuclear factor erythroid 2-related factor 2 (Nrf2) and its downstream targets such as heme oxygenase-1 (HO-1), NAD(P)H quinone dehydrogenase 1 (NQO-1), catalase (CAT), glutathione peroxidase (GPx), superoxide dismutase (SOD), and glutamate–cysteine ligase catalytic subunit (GCLC), while decreasing expression of pro-oxidant enzymes such as inducible nitric oxide synthase (iNOS) and gp91-phox.

It has been suggested that repeated HBOT exposures might lead to increased antioxidant protection but not to an accumulation of oxidative damage, as it has been shown that HBOT-induced DNA damage can only be detected immediately after the first, but not after subsequent, HBOTs [14,112]. Repeated intermittent hyperbaric oxygen exposures can enhance antioxidant defenses through adaptive mechanisms. In an in vitro study conducted with lymphocytes from patients with type 1 diabetes mellitus, HBOT was shown to reduce iNOS expression and consequently NFkB levels [78,113]. The effect of HBOT on oxidative stress balance and mitochondrial properties is shown in Figure 4.

A more recent study investigating the effect of HBOT on the cardiovascular system and oxidative stress in diabetic Wistar albino rats has shown that HBOT has no pro-oxidant effect [114] and that the recruited antioxidant enzyme system has a protective effect against oxidative damage [115], as it has been previously suggested that HBOT upregulates antioxidant gene expression in human endothelial cells, decreases ROS production, and protects against oxidative damage [9,86,106,107].

## 5. Effects of HBOT on Endothelial Dysfunction and Cardiac Function

A single HBOT [116] and a single exposure to normobaric hyperoxia [117] can lead to increased peripheral vasoconstriction and increased afterload, induce bradycardia [118,119], and reduce left ventricle systolic function [120], with cardiac output likely to be reduced via a baroreflex-mediated mechanism triggered by vasoconstriction [118].

In contrast to a single exposure, intermittent HBOT increases HIF-1α activity, VEGF expression, stem cell proliferation, and the level of endothelial progenitor cells that induce angiogenesis and improves oxygenation in the ischemic region [12,99,120,121]. In addition to improved tissue oxygenation and increased blood flow in the ischemic area of the myocardium, HBOT has been shown to improve aerobic metabolism and myocardial energy metabolism, decrease heart rate and myocardial contractility resulting in lower oxygen consumption, and alleviate symptoms of myocardial hypoxia [98,122]. Bennett et al. reported that HBOT can reduce myocardial ischemia and mortality in patients with acute coronary syndrome [123]. A study conducted in rats showed that HBOT can significantly improve the efficacy of myocardial infarction treated with stem cell transplantation due to increased myocardial oxygenation, enhanced expression of VEGF, and cardiomyocyte recovery [124].

HBOT has been shown to significantly increase mRNA and protein expression of eNOS [125] and increase NO levels [126], thus having a protective effect on endothelial dysfunction [127]. In addition, HBOT decreases the expression and activity of iNOS, an important link between metabolic dysfunction and inflammation [128], through mechanisms involving ERK1/2, Akt, and NFκB [113], thus reducing inflammation and subsequent progression of atherosclerosis [86,113]. As all risk factors for atherosclerosis are interrelated, the potential effect of HBOT on endothelial function and vascular reactivity modulating vasodilator and vasoconstrictor production may explain the positive effect of HBOT on atherosclerotic changes, glycemia, and inflammatory markers, as previously suggested [129].

HBOT has been demonstrated to promote collateral circulation [130], inhibit the development of atherosclerosis [131], improve endothelial function in patients with slow coronary flow [122,132], increase the expression of various fibrinolytic factors, and reduce blood viscosity [133]. In addition, HBOT has been demonstrated to improve myocardial perfusion, reduce inflammation and vascular endothelial dysfunction, and improve myocardial microcirculation in patients after implantation of drug-eluting stents [134], as well as to improve myocardial diastolic function in diabetic patients [135]. A study investigating the effect of HBOT on echocardiographic parameters in asymptomatic patients showed an improvement in mitochondrial functions after a regenerative HBOT protocol, an improvement in left ventricular function, and better cardiac performance [98], which is related to the better availability of oxygen to myocyte mitochondria and improved mitochondrial functions as demonstrated in previous studies [136,137,138].

## 6. Effects of HBOT on Atherosclerosis and Plaque Stability and Endothelial Integrity

HBOT has shown potential benefits in improving plaque stability and maintaining endothelial integrity, both key factors in the treatment of cardiovascular disease, particularly atherosclerosis [139]. The stability of the atherosclerotic plaque is important to avoid rupture, which can lead to acute cardiovascular events such as myocardial infarction or stroke. Increased oxygen levels following HBOT promote a reduction in oxidative stress and inflammation, which are two major causes of plaque instability. Some studies suggest that HBOT may reduce ROS levels and inhibit inflammatory cytokines involved in plaque destabilization, including TNF-α and IL-6. This reduction in oxidative stress serves to stabilize the extracellular matrix and reduce the risk of plaque rupture due to reduced activity of MMPs [86,140,141]. In addition, endothelial and vascular smooth muscle cells are stimulated by the hyperoxic state induced by HBOT, which stimulates autophagy. This cellular process contributes to the removal of damaged components and the maintenance of vascular health, thus contributing to plaque stability [142]. More importantly, autophagy plays an important role in the maintenance of cellular homeostasis, a critical condition for the prevention of further endothelial damage or plaque formation/progression [141,143].

As the maintenance of endothelial integrity is important for the regulation of vascular tone and the prevention of thrombosis, it is an important factor in the maintenance of overall cardiovascular health. HBOT has been shown to improve endothelial function by increasing the bioavailability of NO, which is considered one of the main molecules involved in vasodilation. Increased NO levels improve blood flow and reduce vascular resistance, which may help maintain endothelial health [125,126].

HBOT also promotes angiogenesis, or the development of new blood vessels, by increasing the expression of involved growth factors such as VEGF. This in turn should promote further repair and regeneration of the damaged endothelium and improve the overall integrity of the vascular lining [14,86,144,145]. In addition, HBOT was associated with a decrease in endothelial cell apoptosis and an increase in endothelial progenitor cell activity, which further supports endothelial repair [13]. There is evidence that HBOT promotes recovery of the endothelial glycocalyx by reducing oxidative stress and promoting cellular regeneration [81,146].

HBOT protects the glycocalyx by reducing two of the main causes of its degradation: oxidative stress and inflammation. The procedure increases tissue oxygen levels, which in turn increases the activity of antioxidant enzymes such as SOD and reduces the negative effects of ROS on the glycocalyx. This helps to maintain the structural integrity of the glycocalyx and avoid an inflammatory cascade leading to endothelial dysfunction and plaque formation [15,46].

HBOT enhances both plaque stability and endothelial integrity by reducing oxidative stress, increasing NO bioavailability, and stimulating cellular repair mechanisms. HBOT is an attractive adjunctive therapy for the treatment of cardiovascular diseases characterized by endothelial dysfunction and plaque instability. It is important to emphasize that HBOT contributes to the overall stability of the endothelial barrier by supporting the glycocalyx: it reduces vascular permeability and inflammation and slows down the process of atherosclerosis development. This makes HBOT a great supportive therapy for cardiovascular health, especially in conditions where the glycocalyx is significantly damaged, such as diabetes, hypertension, and chronic inflammation [86,139,140,141,142,143,144].

## 7. The Effect of HBOT on Cardiovascular Risk Factors—Hypertension, Aging, Obesity, and Glucose Metabolism Regulation

Cardiovascular risk factors promoting atherosclerosis, such as hypertension, diabetes, aging, and obesity, are associated with damage to the endothelial glycocalyx. Vascular stiffness, an independent predictor of cardiovascular risk, is the result of damage and thinning of the endothelial glycocalyx induced by hypertension and vascular aging [147] and develops in hypertensive patients because of degeneration of the extracellular matrix of elastic arteries [56,60]. In a mouse model of age-related vascular stiffness, endothelial dysfunction was associated with low glypican-1 levels and endothelial dysfunction [148].

Hypertension is both a cause and a consequence of endothelial dysfunction. Endothelial dysfunction is considered one of the most important pathophysiological components of hypertension and is characterized by an imbalance between vasodilatory substances such as NO and vasoconstrictive substances such as endothelin-1 [149], which leads to increased vascular tone and resistance, resulting in self-perpetuating hypertension. Endothelial dysfunction in hypertension is associated with structural changes in the vasculature, including increased arterial stiffness, which in turn contributes to increased blood pressure and cardiovascular risk [150,151,152].

High blood pressure exerts shear stress on the endothelium, causing mechanical injury and overproduction of vasoconstrictors, including endothelin-1, while simultaneously reducing the availability of NO [149,150]. Oxidative stress is a feature of hypertension and, in turn, leads to a further deterioration of endothelial function. In hypertension, the enzymatic activity of eNOS is impaired due to oxidative stress caused by ROS, leading to reduced formation of NO, promoting vasoconstriction and inflammation, and further exacerbating endothelial damage [153,154].

Improved bioavailability of NO through measures such as antioxidant therapy and lifestyle modification may restore endothelial function [155,156]. Pharmacologic treatments such as angiotensin-converting inhibitors and angiotensin II receptor blockers are commonly used to improve endothelial function, reduce oxidative stress, and promote vasodilation. Drugs such as ramipril and telmisartan have been shown to improve endothelial function by increasing NO availability and reducing inflammation in hypertensive patients [157,158]. Exercise is also considered an effective non-pharmacological approach to improve endothelial function. Regular exercise, including aerobic and resistance training, improves vascular shear stress and thus stimulates NO production, improving vascular reactivity. This leads to improved vasodilation and a reduction in blood pressure [150,158]. The increased NO bioavailability and improved vascular relaxation in association with a decrease in ROS provided by HBOT suggest a positive effect on vascular dysfunction and high blood pressure [15]. On the contrary, a recent systematic review analyzing the results of randomized clinical trials [159] reported an increase in blood pressure following HBOT [160], as did a recent study [161], while another study reported one case of hypotension [162], suggesting further studies are needed to evaluate possible HBOT effects on blood pressure regulation.

In addition to disease-related changes in the cardiovascular system, vascular aging is a physiological process characterized by microvascular dysfunction, impaired perfusion, and reduced capillary density [163]. The decrease in thickness and integrity of the endothelial glycocalyx with age has been confirmed in both mice [164] and humans, with an average 30% reduction observed in 60-year-olds compared to 30-year-olds [165]. In addition, aging has been shown to alter the amount of HS on the glycocalyx surface [166] and the fine structure affecting the HS sulfation process [167], thus reducing the interaction with plasma proteins, which impairs hemostasis [15,167].

Age-related damage to the endothelium decreases NO production and leads to increased blood flow resistance, decreased perfusion, and increased cardiac afterload [168]. With increasing age, the contractility of the left ventricle decreases, and the ejection fraction and secondary afterload decrease due to the increasing afterload [169,170]. A prolonged HBOT protocol has been demonstrated to increase left and right ventricular systolic function and improve myocardial performance in elderly patients [98]. In addition, HBOT has been shown to improve echocardiographic parameters of the aging heart in asymptomatic individuals [171].

Aging of the heart leads to declining function and increasing susceptibility to disease and is strongly associated with pulmonary dysfunction [171]. Although the different HBOT protocols used in numerous studies have led to conflicting results, accumulating evidence from animal and human studies suggests that HBOT, when used appropriately, has a positive effect on the aging heart and can counteract the age-related decline in myocardial and cardiopulmonary function [14,170,171].

HBOT has been shown to attenuate the exacerbation of cardiac dysfunction, restore the expression of cardiac senescence markers, and improve the parameters of cardiac function in D-Gal-induced aging rats [172]. In addition, 60 repeated daily sessions at 2 ATA of HBOT were shown to improve left and right ventricular systolic function and improve cardiac performance in asymptomatic elderly patients but did not induce significant changes in diastolic parameters [135]. It has been shown that a single exposure to HBOT leads to a slight improvement in diastolic function with a negative trend in cardiac systolic function [173] and improves myocardial diastolic function in elderly diabetic patients [135]. In addition, repeated intermittent exposure to HBOT has been shown to improve exercise capacity in aging adults and cardiac perfusion has been shown to be a significant mechanism associated with the observed improvements [169]. During the aging process, there is a decrease in cardiac mitochondrial functions [172,174]. In animal and human studies, HBOT has been shown to improve mitochondrial functions [136,137,138], which is associated with beneficial effects on the heart [98]. Moreover, a recent study in diabetes-induced rats has shown that the combined treatment with HBOT and insulin has a beneficial effect on the chromodynamics of the isolated rat heart by improving cardiodynamic parameters describing systolic and diastolic cardiac function and coronary flow [114], as shown in similar previous studies [175,176].

In cardiometabolic diseases, insulin resistance and alterations in glucose and lipid homeostasis contribute to endothelial dysfunction that develops because of increased oxidative stress, reduced NO production, and increased secretion of adipokines, endothelin-1, and fibroblast growth factor 2, which stimulate inflammation, intimal growth, angiogenesis, and smooth muscle cell proliferation [177,178]. Insulin resistance is considered a key factor in the development of endothelial dysfunction [177] and metabolic syndrome, a pathological condition that includes hypertension, central obesity, and atherogenic dyslipidemia [177]. In addition to damage to the endothelial glycocalyx, obesity has been shown to lead to a loss of flow-induced vasodilation due to a decrease in NO production and NO availability [15,179]. Recently, studies have shown that the impairment of flow-mediated vasodilation and endothelial dysfunction in obesity varies between different vascular beds and was observed in visceral adipose arteries but not in subcutaneous adipose arteries, where endothelial function remained normal [180]. The glycocalyx could be functionally and structurally different in different organs [15], as shown by a study in obese mice suggesting that a thicker glycocalyx in brain vessels could have a protective effect [181].

Insulin resistance is associated with increased release of circulating free fatty acids (FFAs) from adipose tissue [182], which inhibit insulin-mediated glucose uptake in muscle, leading to hyperglycemia and hyperinsulinemia. In addition, FFAs increase glucose, triglyceride, and very-low-density lipoprotein (VLDL) production and reduce conversion to glycogen [177,183]. Hypertriglyceridemia leads to a reduction in protective high-density lipoproteins (HDLs), while the relative depletion of unesterified and esterified cholesterol and phospholipids increases the formation of low-density lipoproteins (LDLs), which have proatherogenic properties [184].

In animal models, HBOT has been shown to improve glucose metabolism by increasing oxidative capacity and GLUT4 expression in skeletal muscle [185] as well as brown adipose tissue volume and thermogenesis [185,186]. A recent study suggested a potential benefit of HBOT and insulin treatment in the management of oxidative stress and cardiovascular complications in diabetic patients [114]. Studies conducted in rats with metabolic syndrome have shown that HBOT [187,188] and exposure to mild hyperbaric oxygen [189] improved insulin sensitivity and biochemical parameters of dyslipidemia. HBOT has been shown to reduce hyperlipidemia, promote weight loss in rats [190], and decrease body weight and abdominal fat in rats with metabolic syndrome [187] but showed no effect on lipid profile or body weight in aging or age-obese rats [172].

In cardiovascular disease and diabetes mellitus, glycocalyx degradation leads to endothelial dysfunction due to increased oxidative stress, inflammation, and impaired NO signaling [191]. Hyperglycemia is one of the most important factors for endothelial glycocalyx damage in diabetes mellitus [192]. This is due to specific molecular mechanisms that include insulin resistance, high circulating glucose levels, the formation of advanced glycation end products (AGEs), eNOS uncoupling, and a decrease in eNOS activity [15,193,194]. Hyperglycemia induces the formation of AGEs, which react with proteins in the glycocalyx, leading to its thickening and dysfunction. AGEs bind to receptors for AGEs (RAGEs) on endothelial cells, activating molecular pathways that lead to oxidative stress and inflammation. The formation of ROS leads to direct oxidative damage to glycocalyx components, impairs endothelial barrier function, and promotes vascular leakage, which contributes to glycocalyx degradation [195].

Chronic low-grade inflammation contributes to the degradation of the glycocalyx and increases endothelial permeability in diabetes mellitus [194]. High glucose levels induce inflammatory stimuli [196] and increase the synthesis of pro-inflammatory cytokines [197], particularly TNF-α, that trigger the production of MMPs, such as MMP-2 and MMP-9, leading to vascular remodeling and inflammation and eventually to macrovascular disease [194]. These enzymes degrade glycocalyx leading to increased vascular permeability and leukocyte infiltration. Hyperglycemia enhances the expression of endothelial adhesion molecules and selectins, which facilitate the binding of leukocytes to the endothelial surface and initiate a cascade of inflammatory events that lead to further destruction of the glycocalyx [198]. Recently, it has been shown that treating low-grade inflammation in diabetes can help improve cardiovascular health [199]. Endothelial cell apoptosis is another important component of hyperglycemia-induced glycocalyx damage [200] that leads to vascular complications such as diabetic microangiopathy [201].

As suggested by a recent systematic review [202], HBOT has been shown to affect glucose metabolism by increasing insulin sensitivity, decreasing serum insulin, and decreasing HbA1C [203,204,205]. Although there are discrepancies in the results obtained, mainly due to different HBOT protocols, study durations, or treatments, HBOT has been shown to significantly reduce glucose levels [206,207] and improve insulin sensitivity [203,204,205] with a reduction in basal glucose levels after several HBOT sessions [160,203].

iNOS is considered an important link between metabolic disorders and inflammation [95,128], and after induction, it produces 100- to 1000-fold more NO than NOS [208], leading to the biosynthesis of peroxynitrite, tissue damage, and reduced bioavailability of NO [113]. Hyperglycemia can increase NO production through increased expression of eNOS and iNOS genes and proteins [209], but mainly through the activation of iNOS [210], and it is associated with vascular complications [113,211]. HBOT has been found to decrease the activity and expression of iNOS, followed by a decrease in NO production [113,208], reducing inflammation and the subsequent progression of atherosclerosis [113].

In type 2 diabetes mellitus, insulin resistance impairs the normal vasodilatory function of the endothelium and contributes to glycocalyx damage by interfering with insulin-mediated protective mechanisms on endothelial cells. Normally, insulin promotes NO production via the PI3K-Akt-eNOS pathway, but in type 2 diabetes mellitus, this pathway is impaired, reducing NO availability and leading to vasoconstriction [212]. Hyperglycemia contributes to a decrease in NO bioavailability due to increased oxidative stress and ROS production as well as decreased endothelial eNOS activity. Decreased NO levels can lead to impaired vasodilation and increased vascular stiffness, which exacerbates the vascular complications of diabetes [213].

Insulin resistance and hypothyroidism have been associated with decreased eNOS activity and an increased risk of cardiovascular disease [59,214]. The influence of hormones such as insulin, thyroid hormones, and estrogen on eNOS expression [59,215,216] may partly explain the differences in the incidence of cardiovascular disease between men and women. High estrogen levels have been associated with a lower risk of cardiovascular disease in women up to menopause [216], as it inhibits the development of atherosclerosis by stimulating eNOS expression and activity [59,216,217].

## 8. The Effect of HBOT on Inflammation in Cardiovascular Disorders

Degradation and shedding of the endothelial glycocalyx is considered an early marker of endothelial injury [218,219]. Upon injury, damage and shedding of the glycocalyx lead to increased leukocyte rolling and adhesion [20,35], impaired vascular tone [15,16,17], impaired coagulation [19], and increased vascular permeability [36], while cleaved glycocalyx components induce dendritic cell activation and cytokine secretion [37].

Soluble HS and small glycocalyx fragments stimulate inflammation through toll-like receptor (TLR) signaling, activating the transcription factors NFκB, c-Jun amino N-terminal protein kinase (JNK), and activator protein-1 (AP-1), as well as the transcription of genes involved in the immune response, leading to the induction of cytokine synthesis, such as TNF-α, IL-1β, IL-2, IL-6, IL-8, and IL-10, and the chemokine macrophage inflammatory protein-2 (MIP-2), keratinocyte-derived chemoattractants, RANTES, and monocyte chemoattractant protein-1 (MCP-1) [37,220], and finally the activation of T cells, vascular dysfunction, and ischemia–reperfusion injury [37,220,221,222]. In addition, soluble syndecan-4 increases the expression of adhesion molecules and IL-1β and TNF-α on cardiomyocytes, thereby increasing inflammatory recruitment and stimulating the inflammatory response [223]. In addition to stimulation of the immune response, soluble glycocalyx components can inhibit the inflammatory response. Soluble HS can inhibit the cytokine IL-10 and interact with leukocytes, blocking their adhesion to endothelial cells [224]. Syndecan-1 has been shown to reduce neutrophil accumulation [225] and inhibit the expression of IL-1β, IL-6, and TNF-α, as well as the activity of the same pro-inflammatory chemokines CCL7, CCL11, and CCL17 [226].

Pro-inflammatory cytokines have been found to inhibit the production of NO, impair endothelium-dependent dilatation [227], and alter myocardial perfusion [228], particularly during the inflammatory response following cardiac surgery [229]. Pro-inflammatory IL-1β and TNF-α can impair vasoregulation [227] and impair endothelial relaxation [229]. Endothelial dysfunction may contribute to the development of stenosis at the graft anastomoses due to acceleration of the atherosclerosis process or medial hyperplasia, thus decreasing the long-term success of cardiac surgery [62]. The onset of bypass has been associated with an immediate disruption of microcirculation due to an acute reduction in capillary density [230], which may persist for the first three postoperative days [219,230,231]. Damage to the endothelial glycocalyx during cardiopulmonary bypass (CPB) could lead to detachment and increased numbers of dysfunctional circulating endothelial cells [232], which could act as non-professional antigen-presenting cells presenting glycocalyx degradation products to memory effector T cells [233], resulting in endothelial cell damage during CPB [234,235].

HBOT has been shown to have an immunomodulatory effect on various inflammatory cells and may potentially contribute to the prevention and treatment of cardiovascular disease since the chronic, low-grade inflammatory state associated with aging plays a significant role in the pathogenesis of these age-related diseases such as cardiovascular disease [14,15]. Exposure to hyperbaric oxygen has been shown to induce apoptosis of lymphocytes [236] and neutrophils [237], reduce neutrophil recruitment and activation [238], reduce ROS production, activate MAPKs, and release neutrophil extracellular traps (NETs) [238,239]. In cells from healthy volunteers, HBOT was found to reduce ROS production by neutrophils but had no effect on neutrophil phagocytic activity, cytokines, or systemic oxidative stress [103].

Immunomodulatory effects of HBOT with effects on immune cells, especially Th1 and B lymphocyte subsets [13,240], along with alteration of the CD4+:CD8+ ratio, influence lymphocyte proliferation and the activation of neutrophils in hyperoxic regions [239].

HBOT has been shown to influence the polarization of Th17 cells to T-reg cells and reduce cell hypoxia, thereby alleviating rheumatoid arthritis [241]. In addition, HBOT induces the expression of antioxidants and regulates pro-inflammatory cytokines, thereby reducing chronic inflammation via a direct effect on HIF-1 [242]. Long-term exposure to HBOT has been shown to suppress the development of autoimmune symptoms such as proteinuria, facial erythema, and lymphadenopathy, in addition to increasing survival time and improving immune complex deposition associated with a decrease in inflammatory cells and anti-dsDNA antibody titers [243].

Although there are conflicting results, HBOT modulates the inflammatory process by affecting the expression of cytokines and other mediators, resulting in a general anti-inflammatory state and a decrease in NF-κB, IL-1β, IL-6, and IL-8, as shown in a systemic review [86]. Decreased expression of MMPs after HBOT is also well documented [244,245,246], and this effect on MMP expression is delayed and manifests after two or three HBOT sessions [245].

HBOT has been found to reduce the expression of NF-κB, a transcription factor responsible for the overexpression of mediator proteins, in a sepsis model, neuroinflammation, healthy cells, and cancer cells at the protein level [247], thereby decreasing the production of pro-inflammatory cytokines [86]. Several studies have shown a reduction in various pro-inflammatory cytokines and inflammatory mediators after HBOT, including IL-1β, IL-2, IL-6, TNF-α, IFN-γ, PGE2, and COX-2 [236,248], and increases in anti-inflammatory cytokines, such as IL-1Ra, IL-4, and IL-10 [248,249].

HBOT has been shown to have a protective effect against multi-organ damage following generalized inflammation by interfering with the TLR/NF-κB pathway and downregulating pro-inflammatory cytokine secretion [250]. HBOT was found to increase IL-10 [251] and IL-4 [252] and decrease levels of TGF-β messengers and proteins [86,253]. A systematic review confirmed the increase in IL-4 levels but found no effect on IL-10 and a decrease in TGF-β [86].

HBOT has been shown to reduce inflammation in patients with type 1 diabetes by regulating iNOS activity/expression and nitrite/nitrate production in lymphocytes [113] and inhibit neutrophil adhesion to vascular endothelium as a localized process due to increased activity of NOS and MPO in neutrophils, increased NO release, and excessive S-nitrosylation of β-actin required for β-integrin [254]. Furthermore, HBOT reduces ICAM-1 on the vascular endothelium in patients with sepsis caused by necrotizing soft tissue infections [255] and thus reduces inflammation on the endothelium [256].

## 9. Mechanism of Endothelial Cells and Glycocalyx Damage Injury During Ischemia–Reperfusion Injury in Cardiovascular Disorders

Ischemia–reperfusion injury is defined as a critical injury that occurs after an interruption of blood flow to a tissue, followed by its restoration. This injury involves a multicomponent process that results in cellular damage. Among the most affected cells are the endothelial cells and their protective glycocalyx layer [257]. The glycocalyx functions like a barrier and therefore modulates the interactions between the blood and the endothelial cells, thereby affecting vascular permeability and inflammation [258,259]. Reperfusion leads to a restoration of blood flow and thus to a sudden influx of oxygen, which leads to the formation of ROS, leading to severe endothelial damage and endothelial dysfunction [15,260,261], mitochondrial dysfunction, and an inflammatory response mainly due to activation of the complement system [262].

These ROS can oxidize lipids, proteins, and DNA, causing apoptosis and necrosis of endothelial cells [263]. Ischemia–reperfusion injury also leads to an upregulation of pro-inflammatory cytokines and adhesion molecules, resulting in the recruitment of immune cells to the sites of injury. This inflammatory cascade favors further damage and promotes endothelial dysfunction [264,265]. This restoration of blood flow can disrupt the intercellular junctions of endothelial cells, thereby increasing vascular permeability and leading to edema. Loss of glycocalyx integrity exacerbates this dysfunction by facilitating the penetration of harmful substances through the endothelium [266,267,268]. Research has shown that prolonged ischemia followed by reperfusion can initiate the apoptotic machinery in endothelial cells, stimulating their death with consequent impairment of endothelial integrity. This loss is particularly detrimental to organs such as the heart and kidneys, which rely on the endothelium for proper tissue perfusion [269,270,271].

The resulting lesion not only disrupts the barrier properties of the endothelium but also increases vascular permeability, allowing edema and inflammation to occur in the affected tissue [261,272]. The endothelial dysfunction associated with ischemia–reperfusion injury also plays an important role in microvascular obstruction, which is a limiting factor for the success of reperfusion therapy and increases the risk of further tissue damage. Therapies aimed at restoring endothelial function, including antioxidant treatments and agents that increase NO production, have been shown to reduce some of the injuries associated with ischemia–reperfusion injury [261,273]. In this context, endothelial dysfunction may represent a final common pathway to explain the process leading to atherosclerosis, hypertension, and ischemia–reperfusion injury. This dysfunction promotes disease progression and impairs outcomes through the loss of the endothelium’s ability to regulate vascular tone, prevent inflammation, and maintain vascular integrity. Therapies aimed at improving endothelial function by reducing oxidative stress and improving NO bioavailability hold promise for improving cardiovascular outcomes in patients with these diseases.

Ischemia–reperfusion injury also significantly damages the glycocalyx. The sudden return of blood flow generates a high shear stress that can potentially injure the glycocalyx, disrupting its structural integrity and thus impairing its function as a protective barrier [274]. Reperfusion can lead to the activation of some proteolytic enzymes [275] involved in the degradation of the endothelial glycocalyx during ischemia-reperfusion injury [15,75,276]. Elevated levels of MMP-3 and MMP-9 have been observed in patients with ischemic heart disease and atherosclerotic plaques [277,278]. After cardiac surgery, CPB, and ischemic stroke, increased concentrations of glycocalyx components such as syndecan-1, HS, and hyaluronan have been found in patients’ blood and urine, which are related to the activation of sheddases, heparinase, MMPs, and hyaluronidase [39,279].

Complement activation and endothelial cell interaction with immune cells, like neutrophils, occur as the earliest inflammatory response during ischemia–reperfusion injury [262]. During the neutrophil-mediated immune response, the endothelial glycocalyx can be damaged by enzymatic degradation or oxidative stress [15]. In addition, damage to endothelial cell–cell junctions by elastase, cathepsins, and MMPs released by neutrophils leads to vascular leakage and edema in myocardial infarction [276,280]. Animal studies in mice in which complement receptor 5a was knocked out showed reduced migration of neutrophils into the post-ischemic myocardium and reduced expression of MMP-9 [281]. Activated neutrophils can form NETs, a net-like structure of decondensed chromatin, histones, and cytoplasmic and granular proteins that are released in peripheral vascular disease, myocardial infarction, and stroke [282]. Highly cytotoxic histones released by NETs lead to disruption of the endothelial glycocalyx and microvascular leakage [283], and the amount correlates with infarct size [284].

Animal studies have shown that detachment of the endothelial glycocalyx during ischemia–reperfusion injury increases concentrations of syndecan-1 and heparan sulfate during circulation [285,286]. In an animal model of cardiac ischemia–reperfusion injury, the reduction in glycocalyx thickness has been shown to occur as early as 5 min after reperfusion, leading to reduced vasodilation mediated by NO [287]. Early shedding of syndecan-1 and HS has been observed in patients after reperfusion following cardiac surgery [219,288,289] and detected in survivors of cardiac arrest, patients undergoing coronary artery bypass grafting (CABG), and patients with acute coronary syndrome [148,286]. In patients with ischemic heart disease, an increased serum level of syndecan-1 correlated with the degree of inflammation and leukocyte recruitment [290]. In patients with heart failure with preserved ejection fraction (HFpEF), an elevated serum hyaluronan level was found to be an independent predictor of worse clinical outcomes [291]. In patients with ischemic stroke, an increase in 3 different GAGs, including HS, keratan sulfate, chondroitin sulfate, and 3 different PGs, including C44, syndecan-2, and syndecan-3, was found 1 week after the incident [292].

This degradation deprives the glycocalyx structure of its protective function and increases vascular permeability [293]. Several studies have concluded that HBOT can restore endothelial function and repair the glycocalyx by promoting the production of endothelial progenitor cells and improving the integrity of the glycocalyx [294,295,296]. A mechanistic understanding of the damage to endothelial cells and their glycocalyx in the context of ischemia–reperfusion injury is necessary to develop appropriate therapeutic strategies. Hyperbaric oxygen therapy may hold promise for mitigating these injuries and restoring endothelial function, warranting further investigation.

## 10. Ischemia–Reperfusion Injury and HBOT

HBOT has emerged as a promising strategy in the mitigation of ischemia–reperfusion injury. By applying 100% oxygen under pressure, HBOT increases the availability of oxygen in ischemic/hypoxic tissues, improving tissue survival, wound healing, and angiogenesis, Hyperoxigenation causes vasoconstriction that reduces tissue edema and improves microcirculation [297,298]. Restored oxygenation restores cell function and cellular energy production, reduces oxidative stress, apoptosis, and ROS toxicity [297,298], and preserves the integrity of the endothelial glycocalyx. These benefits make HBOT a useful tool in the treatment of ischemia–reperfusion injury. Due to differences in ischemia–reperfusion injuries, and HBOT protocols, it is difficult to determine the main actors responsible for the beneficial effects of HBOT [86,298]. Among other outcomes, HBOT has a significant immunomodulatory effect by inhibiting the effects of neutrophil adherence to the damaged vessels which results in protease activity and ROS production, vasoconstriction, reduced blood flow, and additional tissue destruction. Therefore, HBOT attenuates reperfusion injury by inhibiting neutrophil adherence to damaged vessels [299].

Accumulating evidence suggests a positive effect of HBOT in the preconditioning of patients following elective CABG. Initial animal studies have shown that pretreatment with HBOT can induce tolerance to central nervous system ischemia and reduce infarct volume and functional deficit [300,301].

Alex et al. [302] showed in a double-blind study that preconditioning patients with HBOT, before on-pump CABG improved left ventricular function, reduced myocardial damage and intraoperative blood loss, shortened intensive care unit length of stay, and reduced postoperative complications and incidence of infection in sternal wounds after CABG, probably due to a prophylactic antimicrobial effect. It was reported that HBOT preconditioning in patients scheduled for elective on-pump or off-pump surgery and CABG had both cerebral and cardiac protective effects [301,302]. In addition, preconditioning of patients undergoing elective on-pump CABG for the first time with three courses of HBOT administered 24, 12, and 4 h before surgery improved myocardial function, shortened the length of stay in the intensive care unit, and reduced postoperative complications [130].

In a 2021 review, Zhang et al. found that the administration of HBOT in patients undergoing CABG significantly reduced the incidence of perioperative myocardial infarction and improved overall cardiac function. The authors also emphasized that protocols should be standardized to optimize the use of HBOT in patients undergoing cardiac surgery [303,304].

A randomized controlled trial study found significant improvements in end-systolic volume index and ejection fraction in the HBOT-treated group and a halt to further deterioration of diastolic function in patients with a first myocardial infarction [305]. Also, HBOT is associated with an improvement in perfusion and an increase in ejection fraction following percutaneous coronary intervention in ST-elevation myocardial infarction (STEMI) patients [306].

Clinical trials related to stroke have shown that HBOT, when administered within 24 h of ischemic stroke onset, improves neurologic outcomes and reduces infarct size. Thus, it appears that HBOT can be used as a neuroprotective strategy in the critical early phases of stroke treatment [307,308].

The Cochrane review by Bennett et al. [309] systematically investigated the potential role of HBOT in patients with acute coronary syndrome (ACS), which includes acute myocardial infarction and unstable angina. Their results suggest that HBOT may provide significant clinical benefits by reducing the risk of mortality and major adverse cardiac events (cardiac death and target vessel revascularization by surgical or percutaneous intervention). This reduction in adverse outcomes is likely due to the ability of HBOT to improve myocardial oxygenation in ischemic tissue. In addition, the ability of HBOT to modulate oxidative stress, inflammation, and endothelial dysfunction suggests a broader application in the treatment of ischemia–reperfusion injury associated with ACS. On the other hand, the work of Xuezheng et al. aimed to evaluate the efficacy and safety of supplemental HBOT in acute ischemic stroke and found no causal relationships supporting the use of HBOT to improve clinical outcomes in acute ischemic stroke [310]. Therefore, the potential of HBOT to provide clinical benefits in the treatment of acute myocardial infarction and ischemic stroke should be further investigated through well-designed, large-scale trials to determine its efficacy, optimal therapeutic windows, and integration into current treatment protocols.

Although the evidence for the use of HBOT in ischemia–reperfusion injury is convincing, it must be emphasized that further studies are needed to determine the standardization of treatment protocols, optimal timing, and dosing of HBOT. In this way, future research should also identify subgroups of patients who would best benefit from this therapy. The abundant clinical evidence supporting the use of HBOT for ischemia–reperfusion injury continues to hold promise for improving outcomes following various medical scenarios such as surgical recovery, acute limb ischemia, cardiac ischemia–reperfusion injury, stroke, and organ transplantation. The ever-growing literature points to a very important role of HBOT in the treatment of ischemia–reperfusion injury as an adjunct to traditional treatment modalities.

## 11. Endothelial Dysfunction and HBOT in Chronic Venous Disease

Pro-inflammatory and pro-thrombotic environment and endothelial dysfunction have been associated with atherosclerosis as well as chronic venous disease (CVD) [311,312]. CVD is a condition of multifactorial etiology and pathophysiology, including genetic and hormonal influence, extracellular matrix (ECM) imbalance, and endothelial dysfunction. It is characterized by venous hypertension that is transmitted to microcirculation, contributing to increased endothelial shear stress leading to glycocalyx damage [311,312]. Chronic inflammation causes localized endothelial activation and endothelial dysfunction via a reduction in the expression of anti-inflammatory agents and stimulation of the expression of pro-inflammatory and pro-thrombotic molecules [311]. ED is associated with vascular tone and shear stress [311]. Significantly, an oscillatory flow in the veins of the lower limbs has been linked to a pro-inflammatory endothelial response, and surgical suppression of the oscillatory component of venous reflux was shown to modulate the inflammatory phenotype [313]. Impaired homeostasis of the ECM contributes to the development of CVD and is regulated by MMPs and tissue inhibitors of metalloproteinases (TIMPs). Especially, MMP-1, MMP-8, ADAM-17, and ADAMTS-4 are linked to chronic or irreversible complications of CVD such as venous leg ulcer (VLU), while TIMP-1 and TIMP-2 tend to decrease during CVD worsening [311].

HBOT is an established adjunctive treatment for peripheral artery disease and diabetic foot ulcers, but accumulating evidence suggests that HBOT may also improve outcomes of VLU patients, especially those undergoing surgery, and reduce ulcer size [314,315,316] by improving tissue oxygenation, increasing angiogenesis, modulating inflammatory responses, reducing edema, and increasing collagen deposition [9,13,79]. The imbalance between MMPs and TIMPs is important in the wound healing process. HBOT inhibits MMP expression [244,245,246], and significantly increases the expression of TIMP2, thus providing additional inhibition of MMP activity and influencing the wound healing process. No influence on TIMP 3 and TIMP 4 has been detected [317].

Due to the complexity of multifactorial etiology and pathophysiology, treatment of CVD includes different strategies to maximize the clinical management including compression therapies, interventions directed to control venous insufficiency, and pharmacological treatment directed to specific pathophysiological mechanisms involved, displaying anti-inflammatory, endothelial-protective, and vasoregulatory effects [311,312]. In addition to the correction of underlying risk factors, VLUs are usually treated with standard treatment including infection control, dressings to provide a moist wound environment according to the clinical findings, debridement, and administration of antimicrobials when necessary [311,312,318]. In treatment-resistant chronic wounds, additional therapeutic options could be administered such as surgical treatment and negative pressure wound therapy. New therapeutic modalities such as platelet-rich plasma and cold plasma, as well as recent development of various biomaterials and skin substitutes for wound coverage, provide a new treatment option for therapy-resistant VLU [311,312,318].

## 12. Conclusions and Future Directions

The promising potential of HBOT as a therapeutic intervention in patients with cardiovascular disease by targeting endothelial dysfunction, a critical factor in pathogenesis, appears to positively contribute to vascular homeostasis through mechanisms that promote angiogenesis, increase NO bioavailability, and support the integrity of the endothelial glycocalyx. The ability of HBOT to enhance endothelial repair processes, stimulate the release of endothelial progenitor cells, and improve NO production may potentially mitigate the progression of atherosclerosis. HBOT shows the ability to stimulate angiogenesis and increase NO bioavailability, which helps to improve vascular function and reduce inflammation. NO is crucial for vasodilation. By promoting NO production and HBOT, vascular resistance is lowered, reducing the risks associated with hypertension and other cardiovascular diseases. Increased NO levels also prevent platelet aggregation and thrombosis, which has additional positive effects on cardiovascular health. HBOT has a positive effect on vascular endothelial cells by promoting their repair processes and supporting the structural integrity of the endothelial glycocalyx. In addition, HBOT has been observed to stimulate the release of endothelial progenitor cells, which play a critical role in maintaining vascular health and repairing damaged endothelium. This is a crucial point in the prevention of endothelial cell dysfunction, which is often associated with cardiovascular disease. Through the controlled increase in ROS and RNS, HBOT triggers an adaptive antioxidant response that upregulates protective enzymes such as SOD and catalase. This response alleviates inflammation by reducing pro-inflammatory markers such as TNF-α and IL-6, which play a role in the progression of atherosclerosis. In addition, by stimulating anti-inflammatory cytokines, HBOT helps to balance the immune response, which is beneficial in chronic inflammation associated with cardiovascular disease. Although the therapeutic effects of HBOT are promising, the potential risks of excessive ROS production during HBOT should not be overlooked. Prolonged or high-intensity HBOT application may result in oxidative damage to cellular lipids, proteins, and DNA, potentially exacerbating endothelial dysfunction and leading to adverse effects such as seizures, pulmonary injury, or oxygen toxicity. In cases where oxidative stress is higher than the organism’s antioxidant defenses, HBOT may also induce mitochondrial dysfunction, which could negatively impact cellular respiration and increase systemic oxidative damage. Understanding these dose-dependent responses and the potential for cellular damage is critical for optimizing HBOT protocols, particularly in patients whose antioxidant capacity may be impaired. Given the potential benefits and risks associated with HBOT, future research should prioritize rigorous studies to standardize HBOT protocols, particularly with regard to session duration, oxygen pressure, and frequency of HBOT treatments. The identification of biomarkers that predict response to HBOT could allow for patient stratification and more individualized treatment approaches. Further studies should explore these predictive biomarkers to improve clinical outcomes. Similarly, longitudinal studies on the impact of HBOT on long-term cardiovascular outcomes would provide important insights into its efficacy and safety. In addition, comparative studies investigating HBOT together with other therapies for cardiovascular disease could help to identify optimal treatment combinations for different cardiovascular diseases. Ultimately, HBOT represents a promising therapy for patients with cardiovascular disease. It offers mechanisms that address both the prevention and management of atherosclerosis, endothelial dysfunction, and inflammatory conditions, but future research is needed to enable personalized and optimized applications of HBOT in cardiovascular healthcare that can make an important contribution to the prevention and management of patients with cardiovascular disease.

## Data Availability

No new data were created or analyzed in this study.
